# Evidence of Calcium Signaling and Modulation of the LmrS Multidrug Resistant Efflux Pump Activity by Ca^2 +^ Ions in *S. aureus*

**DOI:** 10.3389/fmicb.2020.573388

**Published:** 2020-10-22

**Authors:** Amy R. Nava, Natalia Mauricio, Angel J. Sanca, Delfina C. Domínguez

**Affiliations:** ^1^Department of Interdisciplinary Health Sciences, The University of Texas at El Paso, El Paso, TX, United States; ^2^Biology Department, El Paso Community College, El Paso, TX, United States; ^3^Biological Sciences Department, The University of Texas at El Paso, El Paso, TX, United States; ^4^Clinical Laboratory Science Program/Department of Public Health Sciences, The University of Texas at El Paso, El Paso, TX, United States

**Keywords:** efflux pumps, LmrS, prokaryotic calcium transport, calcium homeostasis, phenothiazines

## Abstract

Calcium ions (Ca^2+^) play a pivotal role in eukaryote cell signaling and regulate many physiological functions. Although a similar role for Ca^2+^ in prokaryotes has been difficult to demonstrate, there is increasing evidence for Ca^2+^ as a cell regulator in bacteria. The purpose of this study was to investigate Ca^2+^ signaling and the effect of Ca^2+^ on the *Staphylococcus aureu*s multidrug resistant efflux pump LmrS. We hypothesized that antibiotics act by increasing Ca^2+^ concentrations, which in turn enhance the efflux activity of LmrS. These Ca^2+^ transients were measured by luminometry in response to various antibiotics by using the photoprotein aequorin reconstituted within live bacterial cells. Efflux associated with LmrS was measured by the increase in fluorescence due to the loss of ethidium bromide (EtBr) from both *S. aureus* cells and from *E. coli* cells in which the *lmrs* gene of *S. aureus* was expressed. We found that addition of antibiotics to cells generated unique cytosolic Ca^2+^ transients and that addition of CaCl_2_ to cells enhanced EtBr efflux whereas addition of Ca^2+^ chelators or efflux pump inhibitors significantly decreased EtBr efflux from cells. We conclude that antibiotics induce a Ca^2+^ mediated response through transients in cytosolic Ca^2+^, which then stimulates LmrS efflux pump.

## Introduction

Calcium ions (Ca^2+^) are recognized as key messengers and regulators in nearly all cellular functions of eukaryotic cells (Clapham, 1995; Campbell, 2015, 2018; Carafoli and Krebs, 2016). Ca^2+^ signaling is well understood in mammals and regulates a wide variety of processes ranging from cell fertilization to apoptosis (Rajagopal and Murugavel, 2017; Moretti et al., 2019). Cells respond to environmental stimuli by transient changes in intracellular free Ca^2+^ concentration [Ca^2+^]i, which is utilized by cells to transmit information. Physiological responses also depend on the magnitude, speed and spatiotemporal patterns of the Ca^2+^ signal (Berridge et al., 2003; Zampese and Pizzo, 2012).

In contrast to eukaryotes where the molecular mechanisms for changes in [Ca^2+^]i are well understood, in prokaryotes much work needs to be done. However, there is growing evidence that Ca^2+^ also plays a regulatory role in prokaryotes (Broder et al., 2016; Fishman et al., 2018; Moretti et al., 2019; King et al., 2020). Ca^2+^ ions are involved in numerous bacterial cellular processes including: chemotaxis, transport mechanisms, cell differentiation, virulence, gene expression and others (Fishman et al., 2018; Moretti et al., 2019; Takahashi and Kopriva, 2019; for reviews see Domínguez, 2004; Domínguez et al., 2015). In addition, various Ca^2+^-binding proteins have been identified, which possess calcium binding motifs (Zhou et al., 2013; Sarkisova et al., 2014; Domínguez et al., 2015). Similar to eukaryotes, cytosolic Ca^2+^ homeostasis has been demonstrated in various bacteria and cytosolic Ca^2+^ transients occur in response to stimuli (Torrecilla et al., 2000; Domínguez et al., 2011; Guragain et al., 2013; Zhou et al., 2013; Sarkisova et al., 2014; Bruni et al., 2017; González-Pleiter et al., 2017). During infection and inflammation processes, levels of Ca^2+^ fluctuate significantly, which impact host-pathogen interactions (Van Nhieu et al., 2003; Barrán-Berdón et al., 2011; Broder et al., 2016). However, the role of Ca^2+^ in bacterial pathogenesis is limited and warrants further investigation.

*Staphylococcus aureus* is a versatile pathogen that can cause a wide variety of infections and is able to survive in various environments (Dastgheyb et al., 2015; Li et al., 2015; Onyango and Alreshidi, 2018). The unique adaptability of *S. aureus* makes this organism one of the most problematic bacterial pathogens worldwide (Dastgheyb et al., 2015; Hassan et al., 2015; Kobayashi et al., 2015; Onyango and Alreshidi, 2018). The survival strategies of this organism are diverse and include the ability to replicate in phagosomes, the production of a number of virulence factors such hemolysins, immune evasion factors and resistance to cationic antimicrobial peptides, all of which lead to survival in host cells (Fraunholz and Sinha, 2012; Kobayashi et al., 2015). It is notable that Ca^2+^ plays an important role in *S. aureus* cell adhesion, modulation of biofilm architecture, modulation of α-hemolysin, and autophagy (Thomas et al., 1993; Arrizubieta et al., 2004; Eichstaedt et al., 2009; Fraunholz and Sinha, 2012).

*Staphylococcus aureus* can develop resistance to numerous antimicrobial compounds, including antibiotics and biocides (Esposito et al., 2011; Santos Costa et al., 2013; Conceição et al., 2016; Jang, 2016; Foster, 2017). Although the resistance developed by *S. aureus* strains may be due to different resistance mechanisms, multi-drug resistant efflux pumps (MDREP) play a major role in mediating cross-resistance to antibiotics and biocides (Conceição et al., 2016; Sapula and Brown, 2016; Foster, 2017). In prokaryotes, there are seven classes of MDREP identified: the major facilitator superfamily (MSF), ATP binding cassette (ABC) superfamily, multidrug and toxin extrusion (MATE) family, resistance nodulation division (RND), the small multidrug resistant (SMR) family, the proteobacterial antimicrobial compound efflux (PACE) family, and the p-aminobenzoyl-glutamate transporter (AbgT) family (Webber and Piddock, 2003; Hassan et al., 2013; Santos Costa et al., 2013; Jang, 2016; Schindler and Kaatz, 2016; Chitsaz and Brown, 2017). These transport proteins extrude a wide variety of toxic compounds to the exterior of the cell. Since these transporters have a broad range of substrates, high activity of these pumps can result in the efflux of multiple antibiotics, disinfectants, detergents, dyes, and biocides (Piddock, 2006). Furthermore, efflux pumps play a role in host colonization, virulence and adaptive responses that contribute to resistance during infection (Piddock, 2006; Alcalde-Rico et al., 2016; Jang, 2016; Du et al., 2018). To date, more than twenty putative efflux pumps have been identified in the *S. aureus* genome but few have been characterized. Overexpression of these pumps is proposed to contribute to antibiotic resistance and possibly enhances survival in different environments (Santos Costa et al., 2013; Schindler et al., 2015; Jang, 2016; Sapula and Brown, 2016).

Bacteria respond to environmental stimuli (oxidative stress, cold and heat, changes in pH, salinity and osmotic stress including antimicrobials) by changes in intracellular calcium ion, [Ca^2+^]i, (Knight et al., 1991; Herbaud et al., 1998; Torrecilla et al., 2000, 2001; Naseem et al., 2009; González-Pleiter et al., 2017). Since changes in [Ca^2+^]i are linked to gene expression (Naseem et al., 2009; Domínguez et al., 2011) and virulence (Sarkisova et al., 2005; Broder et al., 2016; Moretti et al., 2019; King et al., 2020), we hypothesize that when *S. aureus* cells are exposed to antibiotics or other drugs, they respond by spiking [Ca^2+^]i, which in turn stimulates efflux pump activity. We tested this hypothesis on one of the multi-drug resistant pump proteins of *S. aureus*, the LmrS, by looking at changes in activity of excretion of EtBr from cells upon varying intracellular Ca^2+^ concentrations and by measuring whether the addition of antibiotics to *S. aureus* cells could increase their [Ca^2+^]i concentrations. Using the photoprotein aequorin as a reporter, we demonstrate for the first time, cytosolic free Ca^2+^ concentration changes in *S. aureus* cells in response to stimuli and rates of influx and efflux in live cells. We conclude that cytosolic Ca^2+^ concentration is carefully controlled in *S. aureus*, antibiotics induce a unique response mediated by cytosolic Ca^2+^ and Ca^2+^ ions enhance the efflux of EtBr in the MDREP LmrS.

## Materials and Methods

### Bacterial Strains and Culture Media

*Staphylococcus aureus* ATCC 25923 and clinical isolate EBSA54 were grown in Brain Heart Infusion (BHI) broth (Remel, Thermo Fisher Scientific, Lenexa, KS, United States). *E. coli* strain JM109 containing the expression vector pMMB66EH with the apoaequorin coding sequence was used to conjugate *S. aureus* cells. *E. coli* cells were a gift from Dr. Anthony K. Campbell (Cardiff University, United Kingdom). *E. coli* cells were grown in Luria-Bertani (LB) broth (Becton Dickinson Difco^TM^, Sparks, MD, United States) with carbenicillin (100 μg/ml) (Sigma-Aldrich, St. Louis, MO, United States). Confirmation of *S. aureus* after conjugation was done by culture in Mannitol Salt Agar (MSA) (Becton Dickinson Difco^TM^, Sparks, MD, United States) and testing for coagulase production (plasma rabbit with EDTA, Becton Dickinson BBL^TM^) and latex agglutination (Prolex^TM^ Pro-Lab Diagnostics, Richmond Hill, ON, Canada). Mueller-Hinton broth (Oxoid Ltd., Basingstoke, Hampshire, United Kingdom) was used to determine the Minimum inhibitory concentration (MIC) assays. The vector pRMC2 was purchased from Addgene to clone the amplified *lmrS* gene. *DH5*α *E. coli* cells were obtained from Promega, Madison, WI, United States).

### Chemicals and Biochemicals

Ethidium bromide (EtBr), ethylene glycol-bis (β-aminoethyl ether)-N,N,N′,N′-tetraacetic acid (EGTA), ethylenediaminetetra acetic acid (EDTA), isopropyl-β-D-thiogalactoside (IPTG), 4-(2-hydroxyethyl)-1-piperazineethanesulfonic acid (HEPES) coelenterazine, carbenicillin, CaCl_2_, Carbonyl cyanide m-chloro phenylhydrazone (CCCP), Calmidazolium (CDZ), Verapamil, coelenterazine, Trifluoperazine (TPZ), Chlorpromazine (CPZ), Phosphate Buffer Solution (PBS), Triton X-100, Tris-Acetate EDTA buffer (TAE), and aequorin oligonucleotides were all purchased from Sigma-Aldrich (St. Louis, MO, United States). Taq polymerase master mix for PCR (Promega, Madison, WI, United States), agarose molecular grade and 100 bp molecular ruler were obtained from Bio-Rad, Hercules, CA, United States). Kanamycin, Gentamicin, Streptomycin, Vancomycin, Ciprofloxacin, and Erythromycin (St. Louis, MO, United States). *Bam*HI and *Hin*dIII (Rowley, MA, United States), T4 ligase (Rowley, MA, United States).

### Cytosolic Free Ca^2+^ Measurements: Construction and Expression of Apoaequorin in *S. aureus*

*Staphylococcus aureus* cells were transformed by conjugation using *E. coli* cells containing the apoaequorin coding sequence (apoaequorin is the protein without its prosthetic group, coelenterazine). Conjugation was done according to the protocols for tri-parental mating from The Samuel Miller Lab (2019) Conjugating plasmids into bacteria http://miller-lab.net/Miller Lab/protocols/bacterial-genetics/conjugating-plasmids-into-bac teria/with modifications. *E. coli* and *S. aureus* were grown in 250 mL triple-baffled flasks containing 50 ml LB (Becton Dickinson Difco^TM^) broth with 100 μg/mL carbenicillin (Sigma-Aldrich) and BHI broth (Remel Thermo Fisher) respectively, at 37°C, in a rotatory shaker at 220 rpm overnight. After 18–20 h of incubation, *E. coli* and *S. aureus* cultures were adjusted to an OD_600_ of 0.5. A 1:5 dilution (100 μL of *E. coli* and 400 μL *S. aureus* cells) of the bacterial cultures were incubated in 10 mL of BHI with 100 μg/mL carbenicillin and incubated for 3 h at 37°C (220 rpm). The culture was dotted (25 μl) onto BHI agar plates containing 100 μg/mL carbenicillin (Remel Thermo Fisher; Sigma-Aldrich) and incubated for 48–72 h at 37°C. Transformed *S. aureus* cells were isolated by streaking onto MSA (Becton Dickinson Difco^TM^) for 48–72 h at 37°C. Individual bacterial colonies that fermented mannitol were selected and further tested for coagulase (Becton Dickinson BBL^TM^) and anti-*S. aureus* latex agglutination (Prolex^TM^ Pro-Lab Diagnostics). The presence of the plasmid containing the apoaequorin coding sequence was confirmed by PCR (Bio-Rad iCycler Thermal Cycler, Bio-Rad, Hercules, CA, United States). The oligonucleotides, AQ440LICS: Forward: AAGGAGGAAGCAGGTATGGTCAAGCTTACATCAGACTTC GAC and AQ440LICS-CAS Reverse: GACACGCACGAGG TTTAGGGGACAGCTCCACCGTAG were used for DNA amplification (Domínguez et al., 2011) under the following parameters: Initial denaturation 95°C for 4.0 min, 30 cycles of 95° denaturation for 30 s, annealing at 50°C for 30 s, extension at 72°C for 1 min, and final extension for 10 min at 72°C. The aequorin protein was utilized to monitor the amount of intracellular free Ca^2+^. Aequorin is a protein that luminescence (λ_max_ = 469 nm) when it binds to Ca^2^. The Ca^2+^ concentration of the cytosol was measured at rest (basal levels) and after adding increasing concentrations of CaCl_2_ to the culture media. The luminescence produced is directly proportional to the concentration of free Ca^2+^ within the cytosol.

### Expression and Reconstitution of Aequorin in *S. aureus* Cells

The transformed *S. aureus* were grown in BHI-carbenicillin broth overnight at 37° (220 rpm). Cells were diluted 1:100 in BHI-carbenicillin broth and incubated (same conditions) until the culture reached an OD_600_ of 0.3. The aequorin gene was induced by adding IPTG at 1 mM final concentration and re-incubated for additional 2 h. After 2 h, cells were washed twice with 20 mL of ice cold HEPES buffer (25 mM HEPES, 1 mM MgCl_2_, and 125 mM NaCl, pH 7.0) or at the respective experimental pHs (5, 7, and 9) and pelleted by centrifugation at 4500 rpm for 5 min (Beckman Coulter Allegra, rotor C0650). Bacterial cells were re-suspended in 1 mL of HEPES and 2.5 μM of coelenterazine and incubated in the dark for 1 h at room temperature. After incubation, cells were washed twice first in 20 mL HEPES buffer and resuspended in 1 mL of HEPES. The buffer was adjusted to an OD_600_ 0.4 at the appropriate pH (according to the experiments performed) and stored on ice for subsequent readings or 4°C overnight for stabilization. To address possible contamination from trace amounts of Ca^2+^ and other divalent cations, 0.05 mM of EGTA was added to the HEPES media. All glassware and labware were washed with 0.05 mM of EGTA to rinse off trace levels of calcium from the surfaces.

### Detection and Quantification of Intracellular Ca^2+^

Chemiluminescence was measured using a digital Luminometer GloMax 3000 (Promega) equipped with two dispensers allowing the reading on 96-well Microfluor microtiter plates (Thermo Fisher Scientific). Measurements were done in triplicates on 100 μL aequorin-loaded cells once every 10 s for 60 s to determine resting cytosolic free Ca^2+^ levels. Cells were then injected with CaCl_2_ to a final concentration of 1.0 mM CaCl_2_ and chemiluminescence was monitored for an additional 300–500 s according to the appropriate experimental pHs 5, 7, and 9. At the end of each experiment the remaining amount of aequorin was determined by adding equal volumes of discharge buffer (100 mM of CaCl_2_, 5.0% v/v Triton X-100) as described by Domínguez et al. (2011) to determine the total light output. The total chemiluminescence represented by the available aequorin was used to calculate the concentration of cytosolic Ca^2+^. To determine the possible effect of pH on aequorin activity, cell lysates of *S. aureus*, expressing apoaequorin, were reconstituted with coelenterazine as described before. Aliquots of 100 μl were treated with each chemical using the same concentrations as those used in the treatments (different pHs). After each treatment, aliquots were taken to the luminometer and aequorin was completely discharged by adding an equal volume of 100 mM CaCl_2_ and the discharge buffer to determine the total light output. To rule out the possibility that the luminescence obtained would be due to aequorin being released into the medium by lysed cells or lysis of cells due to addition of calcium, light was measured after addition of Ca^2+^ to the medium in which reconstituted cells were present after removing the cells by centrifugation. We also checked for cell lysis by microscopy. Negative control used for these experiments were cells without the apoaequorin plasmid. Relative light units were converted to μM Ca^2+^ concentrations utilizing a matrix created by Dr. Anthony Campbell [pCa = 0.612(−logk) + 3.745 where k is the rate constant for decay of chemiluminescence (s^−1^)] (Jones et al., 1999).

### Antibiotic Signaling Studies

The antibiotics used for the signaling experiments included: erythromycin, gentamicin, kanamycin, vancomycin, streptomycin, and ciprofloxacin. To determine the MIC for each antibiotic, *S. aureus* cells containing the aequorin plasmid were grown overnight in BHI then subcultured into Mueller-Hinton and grown to an OD_600_ 0.8. The culture was then adjusted to a 0.5 McFarland standard (aprox. 10^8^ CFU/mL) in 5 mL. The MIC was determined using MicroScan autoSCAN-4 automated system using MIC panels for Gram positive bacteria PBCP20. To measure antibiotic stimulus-response, *S. aureus* cells were grown as stated above. *S. aureus* cells containing the aequorin were loaded into a microtiter plate and basal intracellular Ca^2+^ was monitored for 10 s followed by injection of each antibiotic to a final concentration of 0.5 μg/mL in a final volume of 100 μl. Intracellular Ca^2+^ was then monitored for an additional 250 s. We used a standard concentration for all antibiotics to detect unique responses of cytosolic Ca^2+^ transients elicited by each antibiotic. This allowed for elimination of concentration as a variable affecting cytosolic responses.

### Efflux Experiments: Effect of Ca^2+^ and Ca^2+^ Inhibitors in *S. aureus*

Efflux pump activity was measured indirectly and directly in *S. aureus cells*. In the indirect method bacterial cells were pre-incubated in (EtBr) and Ca^2+^ or a Ca^2+^ inhibitor before the beginning of the assay. Fluorescence was monitored after incubation for 20 min. Fluorescence increases as the EtBr accumulates within the cells until it reaches a steady state of efflux/accumulation. In the direct method, bacterial cells were incubated with dye in the presence of an inhibitor such as the proton decoupler CCCP, then washed to remove excess dye and inhibitor. Fluorescence was measured for 5 min after which 1 mM of Ca^2+^ was injected into the media. Efflux was then measured for an additional 20 min. The purpose of this is to observe the rate of efflux of EtBr that is inside the cells and to record the change in slope as they respond to the addition of external Ca^2+^.

Indirect measurements. To determine the effect of Ca^2+^ in the efflux systems of *S. aureus*, EtBr was used as a substrate, and cells were treated with various concentrations of CaCl_2_, Ca^2+^-chelators, and Ca^2+^-dependent inhibitors. The MIC for EtBr was done using the broth dilution method and as recommended by the Clinical Laboratory Standards Institute. A final concentration of 2.5 mg/L EtBr was used for both direct and indirect efflux experiments. Bacterial strains were cultured overnight in BHI broth at 37°C in a Gyromax rotatory shaker (Amerex Instruments) at 220 rpm. Overnight cultures (1:100) were transferred into 250 ml triple-baffled flasks containing 50 mL fresh BHI broth and grown to reach an OD_600_ of 0.8. Bacterial cells were harvested by centrifugation at 5000 rpm for 5 min (Beckman Coulter Allegra, rotor C0650). Cells were washed three times in Phosphate buffer solution PBS (137 mM NaCl, 10 mM phosphate, 2.7 mM KCl) adjusting to the different experimental pHs 5.0, 7.0, and 8.0 (pH for efflux experiments was adjusted at 8.0). Bacterial cell concentration was adjusted to an OD_600_ of 0.6 for each pH. Experimental procedures for EtBr efflux were done following the protocols of Couto et al. (2008), Martins et al. (2011) with modifications. Briefly, fluorescence of EtBr was measured over time to observe the decrease in fluorescence as the cells excreted EtBr. Cells were incubated in 2.5 mg/L EtBr. Bacterial cells in aliquots of 100 μL were placed in 96 microtiter plates and efflux pump activity of EtBr was measured by fluorescence at 585 nm in 96 microtiter fluorescent-based plates (Thermo Fischer Scientific) using a GloMax 3000 Fluorometer (Promega). To assess the effect of Ca^2+^ on efflux, media calcium levels were manipulated by addition of CaCl_2_ (Sigma-Aldrich) to a final concentration of 1.0 and 5.0 mM. Lack of calcium was also investigated by the addition of Ca^2+^-chelators, EGTA, and EDTA (Thermo Fisher), which were added at final concentrations of 5 and 10 mM. To investigate Ca^2+^-dependent transport inhibitors, the phenothiazines, CPZ and TFP (Sigma-Aldrich), were added to final concentrations of 0.1 μM and 30 μM, respectively. The Ca^2+^ channel blocker, verapamil (Sigma-Aldrich) was added at 30 μM. Efflux assays were conducted at the different pHs, 5.0, 7.0, and 8.0. Glucose (0.4%) was used as control for the contribution of metabolic energy to efflux. CCCP (Sigma-Aldrich), a proton uncoupler was used to a final concentration 50 μM to illustrate proton motive force disruption.

Direct measurements. For direct efflux experiments, the cells were grown and cultured as described before. Briefly, the cells were grown overnight in BHI broth at 37°C. The cells were subsequently washed and pelleted twice in PBS and adjusted to a final OD A_600_ of 0.6 and incubated in EtBr at 2.5 mg/L and the indicated Ca^2+^ inhibitor. The cells were then pelleted by centrifugation and the supernatant containing excess EtBr and treatment was removed. The cells were then resuspended in PBS at the appropriate pH. 100 μl were loaded into a 96 microtiter plate and fluorescence was monitored for 5 min at 585 nm to establish basal levels. After 5 min, CaCl_2_ was added to a final concentration of 1 mM and fluorescence was monitored for an additional 20 min.

To investigate if Ca^2+^ had the ability to restore efflux activity inhibited by EDTA, and alkaline pH of 8, *S. aureus* cells were grown overnight in BHI following the methods of Martins et al., 2011. The cells were sub-cultured in a 1:100 ratio and grown for an additional 2 h. The cells were then washed and pelleted twice and re-suspended in PBS at pH 8. The OD A_600_ was adjusted to 0.6 and EDTA and EtBr were added to a final concentration of 5 mM and 2.5 mg/L, respectively. Fluorescence was then measured at 585 nm for 20 min. After 20 min the experiment was stopped and cells were treated with one of the following: CPZ (0.1 μM), CPZ + CaCl_2_ (0.1 μM, 1 mM), and CaCl_2_ (1 mM) in a final volume of 100 μl, loaded to a 96 well microtiter plate. Fluorometry was resumed for additional 20 min.

### Cloning of *lmrS* Gene

To investigate further the effects of Ca^2+^ on an individual *S. aureus* efflux pump, the *lmrS* gene was amplified and cloned into the expression vector pRMC2 (Corrigan and Foster, 2009) using the primers designed by Floyd et al., 2010. Forward: GCAAGCTTATGGCTAAAGTTGAATTAACAAC and Reverse: GCGGATCCTTAAAATTTCCTTCTATTACTTT and transformed into *E. coli* cells strain *DH5-α*. The following thermocycler parameters were used: 95°C 1 min, 35 cycles of 95°C 30 s, 51°C for 1 min followed by 1 min of extension at 72°C and a final extension step for 5 min at 72°C. Direct efflux activity of both *E. coli*-*lmrS* and *E. coli DH5*α was measured as previously described with an EtBr concentration of 1 mg/L. Cells not incubated in EtBr were also used as a control. Briefly, the cells were incubated for 30 min in EtBr and inhibitor. The cells were subsequently pelleted by centrifugation, washed twice and resuspended in PBS buffer. Fluorescence was then measured as described above. After 5 min cells were injected with 1 mM of CaCl_2_. Fluorescence was then monitored for an additional 20 min.

### MIC of the *E. coli-lmrS and E. coli DH5*α

Both the wild-type and *E. coli-lmrS* were grown in LB broth and then subcultured into Mueller-Hinton broth and grown to an OD_600_ 0.8. The culture was then adjusted to a 0.5 McFarland standard (aprox. 10^8^ CFU/mL) The MICs for the antibiotics tested were determined using the MicroScan autoSCAN-4 automated system using PBCP34 panels.

### Statistical Methods

The Kruskal–Wallis test was utilized to determine the differences in Ca^2+^ treatments (CaCl_2_ 0.5, 1.0, and 5.0 mM), Ca^2+^ inhibitor treatments (phenothiazines, verapamil, and Ca^2+^ chelators) compared to controls in fluorescent assays. The same test was used to determine significant differences in intracellular Ca^2+^ levels at concentrations: 0.5 mM, 1.0 mM, and 5.0 mM. To determine significant differences of cytosolic Ca^2+^ at each pH (5.0, 7.0, and 8.0) a one sample *t*-test and Wilcoxon test was conducted. Both the efflux and cytosolic free Ca^2+^ experiments were analyzed using the statistical and graphics software, Graph Pad 8.0.

## Results

### *S. aureus* Cells Maintain Ca^2+^ Homeostasis

**FIGURE 1:**
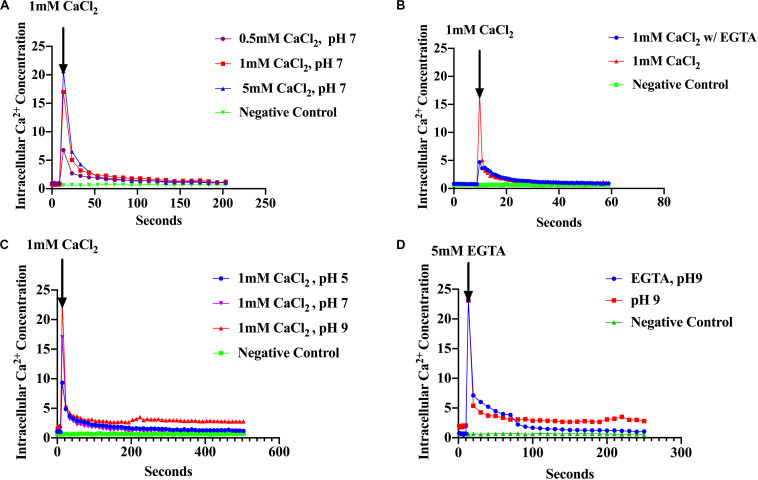
*S. aureus* cells maintain Ca^2+^ homeostasis. **(A)** Cytosolic Ca^2+^ transient in response to various CaCl_2_ concentrations: 0.5, 1.0, and 5.0 mM **(B)** Cytosolic Ca^2+^ transients after addition of 1 mM CaCl_2_ and the Ca^2+^ chelator EGTA **(C)** Cytosolic Ca^2+^ transients in response to different pH 5, 7, and 9 **(D)** Cytosolic Ca^2+^ transients at pH 9 and addition of EGTA. *S. aureus* cells without aequorin were used as the negative controls. The results represent the means of three independent measurements. Significant peak differences were observed in response to pH as well as Ca^2+^ levels. A student *t* was conducted to evaluate treatments (*p* < 0.0001).

The assumption that Ca^2+^ acts as a messenger in prokaryotes is based on the observation that environmental signals induce changes in the level of cytosolic free Ca^2+^. Therefore, measurement of [Ca^2+^]i is essential in establishing that Ca^2+^ might serve as an intracellular signal (Jones et al., 1999; Torrecilla et al., 2000; Campbell, 2015). Based on this premise, we examined the ability of *S. aureus* to maintain Ca^2+^ homeostasis and the response of [Ca^2+^]i to environmental changes such as the presence of antibiotics and pH shifts. We used recombinant *S. aureus* cells constitutively over-expressing the photoprotein aequorin to measure cytosolic free Ca^2+^. Increasing concentrations of external CaCl_2_ (as described in “Materials and Methods”) showed an increase in luminescence directly proportional to the amount of CaCl_2_ injected followed by a rapid decline within seconds (Figure 1A). Conversely, cells treated with Ca^2+^ chelators, such as EGTA showed a sharp decline in cytosolic [Ca^2+^]i as evidenced by low luminescence (all these results were statistically significant) (Figure 1B). These results demonstrate that *S. aureus* maintains cytosolic Ca^2+^ concentrations in the μM range in the presence of CaCl_2_, in the culture broth of 0.5–5 mM, which is consistent with other studies (Jones et al., 1999; Torrecilla et al., 2000, 2001; Domínguez et al., 2011; Guragain et al., 2016). The effect of pH was also examined since *S. aureu*s MFS and MATE transporter families have been shown to use electrochemical gradients and proton motive force as the driving force for extrusion of toxic compounds (toxic compound/H^+^) (Santos Costa et al., 2013; Jang, 2016). External CaCl_2_ was added to a final concentration of 1 mM and the pH was adjusted to pH 5, 7 or 9 (as described in “Materials and Methods”) and luminescence was measured as before. A rapid cytosolic Ca^2+^ transient was observed after injection of 1 mM CaCl_2_ with a fast decline to basal levels. However, cultures maintained at differing pH values had differing cytosolic Ca^2+^ showing different amplitudes (peaks) (Figure 1C). At pH 9, free cytosolic Ca^2+^ rose to 23 μM whereas at pH 7 it was 17 μM and at pH 5 it was 9.5 μM. These results imply that H^+^ is competing for Ca^2+^ influx, perhaps directly at the Ca^2+^ site of the transporter or perhaps by some other mechanism. When EGTA (5 mM) was added after the injection of 1 mM CaCl_2_ the removal of Ca^2+^ caused a sharp decline to basal levels (Figure 1D). *S. aureus* cells without the aequorin plasmid were used as a negative control. Our results indicated that *S. aureus* maintains cytosolic Ca^2+^ in the micro-molar range in the presence of 0.5–5 mM external CaCl_2_. These findings are the first intracellular Ca^2+^ measurements reported in *S. aureus*.

### Unique Ca^2+^ Signals in Response to Antibiotics in *S. aureus* Cells

**FIGURE 2:**
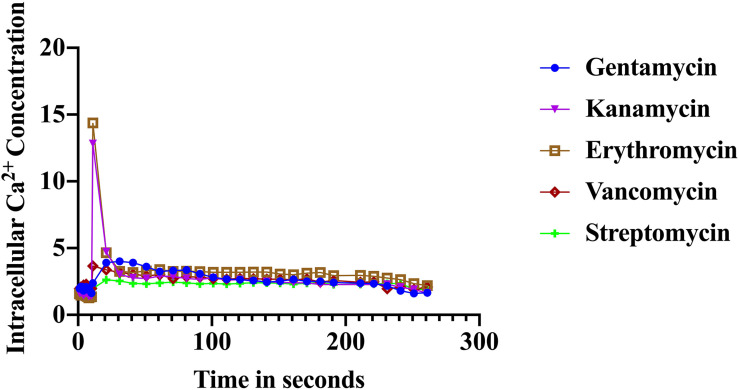
Calcium transients in *S. aureus* cells induced in response to various antibiotics. The results represent the means of three separate determinations.

The immediate response to changes in environmental conditions is crucial for organisms to adapt and survive. Bacteria sense environmental conditions through transients in cytosolic Ca^2+^ (Herbaud et al., 1998; Torrecilla et al., 2000; Naseem et al., 2009). Similar to eukaryotic cells, cytosolic Ca^2+^ transients are very dynamic and vary in shape, amplitude, speed, and spatiotemporal patterns. Several studies showed that exposure to antibiotics have been linked to gene expression including genes related to virulence, biofilm formation, and transporters (Nichols et al., 2011; Romero et al., 2011; Du et al., 2018). Here we investigated the stimulus-response of *S. aureus* cells in sensing the presence of various antibiotics. *S. aureus* cells expressing the photoprotein aequorin were loaded into microtiter plates and basal [Ca^2+^]i was measured for 10 s followed by injection of selected antibiotics (as indicated in the “Materials and Methods” section). Antibiotics used in this study included: erythromycin, gentamicin, kanamycin, vancomycin, streptomycin, and ciprofloxacin. Concentrations of antibiotics were adjusted according to a standard concentration described in “Materials and Methods” section. All antibiotics tested elicited a rapid increase in cytosolic Ca^2+^ (Figure 2). The [Ca^2+^]i response to the antibiotic was very rapid but varied dramatically in amplitude, shape, oscillation pattern, and duration (Table 1) as a function of the antibiotic used. The amplitude of the transient ranged from 12.8 μM Ca^2+^ for erythromycin to 3.4 μM Ca^2+^ for vancomycin. The transient shape differed considerably, some showing oscillations and/or a second peak before reaching basal levels. These results indicate that there is a Ca^2+^ mediated signal response to antibiotics in *S. aureus* and that cells have the ability to differentiate among types of antibiotics, which may trigger further physiological reactions such as gene expression, for cellular adaptation. It is possible that the [Ca^2+^]i response might provide information as to the cells potential to become drug resistant.

**TABLE 1:** Distinct Ca^2+^-mediated signals in response to various antibiotics.

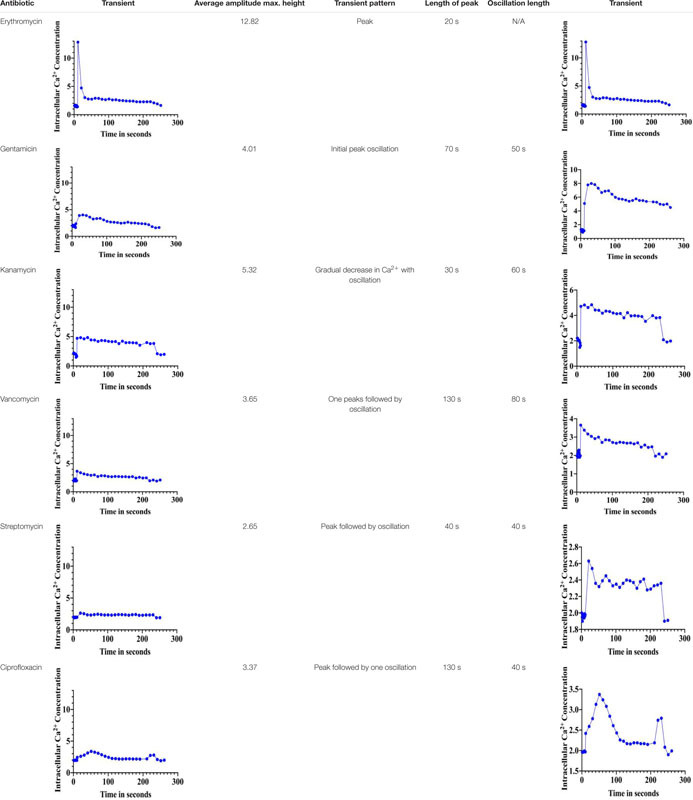

### Effect of Ca^2+^ and Ca^2+^ Inhibitors in *S. aureus* Efflux of EtBr

Measurements of EtBr accumulation were done to assess efflux activity in *S. aureus* cells by fluorescence. The premise is that the higher the efflux, the lower the concentration of EtBr accumulated within bacterial cells resulting in lower fluorescence. Conversely, accumulation of EtBr within cells is indicated by higher fluorescence. Efflux pump activity was measured directly and indirectly in *S. aureus* cells. In the direct method bacterial cells were pre-incubated with EtBr in the presence of an inhibitor of the Ca^2+^ or pump energy source at the appropriate pH, then subsequently washed and fluorescence was measured. In the indirect assays fluorescence was recorded after the pre-incubation period with EtBr and the treatment.

**FIGURE 3:**
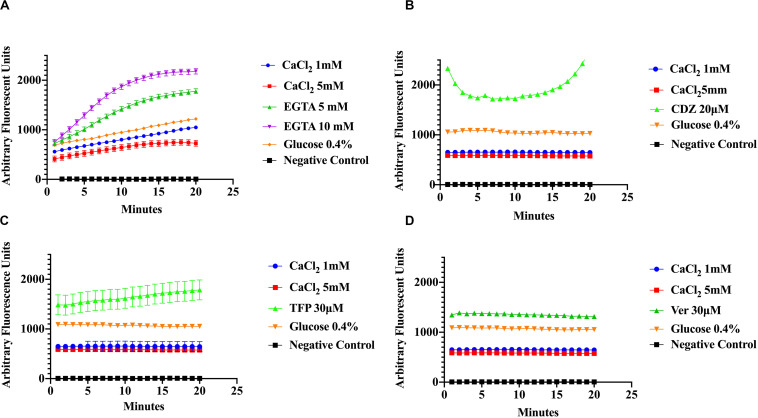
Ethidium bromide accumulation in response to calcium and calcium inhibitors at pH 7. *S. aureus* cells were preincubated in the presence of Ca^2+^ (1 mM and 5 mM), Ca^2+^ chelator EGTA (5, 10 mM), the phenothiazine trifluoperazine (TFP 30 μM), the Ca^2+^-channel blocker verapamil (Ver 30 μM), and the Ca^2+^-Calmodulin inhibitor Calmidazolium (CDZ 20 μM). The results show that calcium enhances the efflux of Ethidium Bromide in *S. aureus* **(A)** effect of CaCl_2_, EGTA, and glucose (4%) on ethidium bromide accumulation at pH 7.6 on *S. aureus.* **(B)** The effect of CaCl_2_, CDZ, and glucose on ethidium bromide accumulation at pH 7 on MRS A cells. **(C)** Effect of CaCl_2_, TFP and glucose on ethidium bromide accumulation (pH 7). **(D)** The effects of Ver, CaCl_2_, and glucose on EtBr efflux (pH 7). The results represent the average of three replicates at each time point. Significant differences were seen between calcium, inhibitor treatments and controls (*p* < 0.0001) Kruskal–Wallis, non-parametric.

**FIGURE 4:**
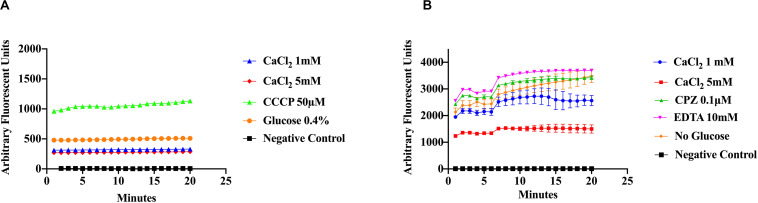
Ethidium bromide accumulation in response to CaCl_2_, Ca^2+^-inhibitors and a proton uncoupler at pH 5 and pH8. Efflux activity of *S. aureus* cells was measured under different pH conditions, calcium and inhibitors. **(A)** *S. aureus* cells were preincubated in the presence of two Ca^2+^ concentrations (1 mM and 5 mM), glucose (0.4%), and CCCP (50 μM) was added to disrupt the electrochemical gradient (pH 5). **(B)** EtBr efflux measured at pH 8, with CaCl_2_ (1 mM and 5 mM), 0.4% glucose, the phenothiazine CPZ (0.1 μ), and EDTA (10 mM). The results represent an average of three replicates. Significant differences were found between the treatments using a non-parametric Kruskal–Wallis test (*p* < 0.0001).

To evaluate the effect of Ca^2+^ on the efflux of EtBr, cells were incubated in various concentrations of CaCl_2_ and EtBr, with or without 0.4% glucose. The addition of glucose was used as a control to distinguish the effects of Ca^2+^ from that of metabolic energy. In addition, samples were incubated in various conditions with one of the following, CaCl_2_, Ca^2+^-chelator EGTA and the phenothiazines, chlorpromazine (CPZ) and trifluoperazine (TFP), which are known to inhibit efflux pump activity in various pathogens including *S. aureus* (Naseem et al., 2008; Koul et al., 2009; Martins et al., 2011; Pule et al., 2016). The phenothiazines are known to bind to Ca^2+^-binding proteins and to target prokaryotic cell membranes (Kaatz et al., 2003; Amaral et al., 2010). These drugs affect Ca^2+^ related processes such as Ca^2+^ binding to transporters and Ca^2+^ dependent enzymes involved in ATP hydrolysis thus inhibiting Ca^2+^ influx and as a consequence, the Ca^2+^ signal. The Ca^2+^ channel blocker, verapamil, which inhibits Ca^2+^ influx/efflux, and the calmodulin inhibitor calmidazolium (CDZ), which is involved in modulation of pumps in eukaryotes, were also used in this study (Couto et al., 2008; Bishai et al., 2013). Efflux activity was monitored at pH 7 according to the protocols of Martins et al., 2011 with modifications. As shown in (Figures 3A–D) addition of EGTA, TFP, Ver, and CDZ showed significant increase in the relative fluorescence (RF), indicating accumulation of the EtBr compared to CaCl_2_ treated cells and control cells (cells without EtBr and cells with EtBr no treatment), which showed low fluorescence. Assays in panel A were conducted at slightly higher (pH 7.6) than panels B-D, showing a slight effect in efflux. A Kruskal–Wallis statistical analysis was used to determine significant differences. Each of the inhibitor treatment was significantly different as compared to 1 mM of Ca^2+^ and to the positive control where the cells were incubated in EtBr alone (data not shown). Each treatment represents the average of three replicates with the mean and ±SD (*p*-value < 0.001). These results show that Ca^2+^ ions have the ability to enhance efflux in *S. aureus* cells. Since proton availability and metabolic energy affect efflux, measurements were also done at different pHs. At pH 5 (Figure 4A) where H^+^ is higher, efflux activity is more efficient compared to efflux at pH 8 where proton availability is lower (Figure 4B).

**FIGURE 5:**
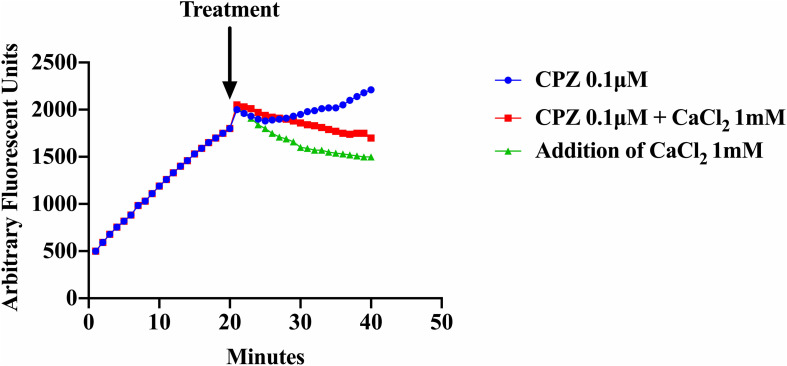
Effect of CPZ, CPZ + CaCl_2_, and CaCl_2_ on the accumulation of EtBr in *S. aureus* cells. Cells were incubated with EtBr and EDTA at a pH of 8 and fluorescence was measured at 585 nm for 40 min. Measurements were halted briefly after 20 min and three treatments were added to a final concentration of 0.1 μM of CPZ, 0.1 μM CPZ + 1 mM CaCl_2_, and 1 mM CaCl_2_ and to a final volume of 100 μl and added to a 96 well microtiter plate. Fluorescence measurements were then resumed for an additional 20 min. The addition of CPZ resulted in further EtBr accumulation (increased fluorescence) whereas the addition of CPZ + CaCl_2_ reduced accumulation of EtBr compared to CPZ alone. The addition of 1 mM CaCl_2_ resulted in the highest increase of efflux (decreased fluorescence) of EtBr. These results represent the average of three independent biological replicates. The results suggest that calcium restored efflux activity in an environment where protons are lost to high alkalinity and metals are chelated from solution.

As shown previously (Figure 3), addition of CPZ, EDTA or EGTA resulted in increased EtBr accumulation in *S. aureus* cells. Here we investigated whether the addition of CaCl_2_ could restore efflux activity suppressed by the Ca^2+^ antagonist CPZ (0.1 μM) and the Ca^2+^ chelator EDTA. We also examined the effect of adding both CPZ and CaCl_2_ together on efflux of EtBr. Our results showed that addition of 1 mM CaCl_2_ decreased the accumulation of EtBr caused by the CPZ. However, when CPZ + CaCl_2_ were added the decrease in fluorescence was less pronounced (Figure 5). These results imply that Ca^2+^ has the ability to restore efflux activity in *S. aureus* cells. These findings are consistent with those reported in *E. coli* (Martins et al., 2011).

### Calcium Enhances Efflux of Ethidium Bromide (EtBr) by LmrS

In order to evaluate the effect of Ca^2+^ in the efflux of EtBr more directly, we cloned the *S. aureus* multi-drug resistant pump *lmrS* gene into *E. coli* cells using the pRMC2 vector (as described in “Materials and Methods” section). The LmrS (lincomycin resistance protein of *S. aureus*) belongs to the MFS superfamily, which transport diverse molecules across the membrane using an electrochemical gradient. The *lmrS* gene was identified by Floyd et al. (2010). The *lmrS* gene was amplified and cloned as described in “Materials and Methods.” To investigate if recombinant *E. coli* cells (DH5α-*lmrS*) have acquired resistance through the incorporation and overexpression of the *lmrS* gene, we evaluated the susceptibility of the recombinant *E. coli* cells to various antibiotics by determining the MIC using the micro broth dilution method (“Materials and Methods”). The results indicate that the *E. coli* cells carrying the *lmrS* gene were resistant to all antibiotic tested in contrast to *E. coli* cells without the *lmrS* gene (Table 2).

**TABLE 2:** Minimum Inhibitory Concentrations (MIC) of cloned *lmrS* vs. control cells.

**Antibiotic**	***LmrS-DH5*α (μ g/ml)**	**Resistant**	***E. coli DH5-*α (μ *g/ml*)**	**Susceptible**
Amoxicillin/Streptomycin (A/S)	18/8	R	8/4	S
Amikacin (AK)	32	R	16	S
Ampicillin (AM)	16	R	8	S
Ceftazidime (CAZ)	16	R	1	S
Cefuroxime (CFX)	16	R	8	S
Cefazolin (CFZ)	16	R	8	S
Ciprofloxacin (CP)	2	R	1	S
Cefuroxime (CRM)	16	R	4	S
Gentamicin (GM)	8	R	4	S
Imipenem (IMP)	8	R	4	S
Levofloxacin (LVX)	4	R	2	S
Piperacillin/Tazobactam (P/T)	64	R	16	S
Tetracycline (TE)	8	R	4	S

### Differences of EtBr Efflux Between *E. coli* DH5α Cloned LmrS (DH5α-LmrS)

**FIGURE 6:**
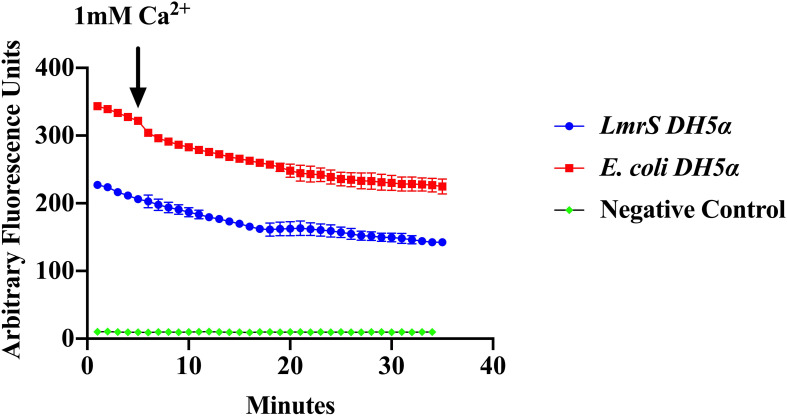
Ethidium bromide efflux measurements in *E. coli* DH5α cells carrying the *lmrS* gene. Direct measurements of EtBr assays in *E. coli* cells carrying the *lmrS* gene and cells without *lmrS* gene (control) were conducted as described in “Materials and Methods.” The results show that the addition of 1 mM CaCl_2_ enhances efflux of EtBr in the cloned *LmrS*, indicated by the lower fluorescence values as well as cells without the cloned *lmrS* gene. Control cells contained no EtBr. Results show the mean of three independent biological replicates. A Kruskal–Wallis test was utilized to detect significant differences in efflux between the *E. coli* DH5α wild-type and the *E. coli* recombinant strain (*p* < 0.0001).

Efflux of EtBr was assessed by direct EtBr assays (“Materials and Methods”). Recombinant *E. coli* cells (DH5α-*lmrS*) were incubated in EtBr (2.5 mg/L). Cells were pelleted and washed twice with PBS and fluorescence was monitored for 5 min at 585 nm. After 5 min 1 mM CaCl_2_ was added and efflux was monitored for an additional 30 min. *E. coli* cells without *LmrS* were also measured as control. Significant differences in efflux (Kruskal–Wallis test) were observed between recombinant *E. coli* cells (DH5α-*lmrS*) and *E. coli* cells (DH5α) without the *lmrS* pump (Figure 6). The expression of *lmrS* significantly increases efflux as indicated by the lower fluorescence values.

### Effect of Ca^2+^ Chelator EGTA and CPZ in Cloned LmrS

**FIGURE 7:**
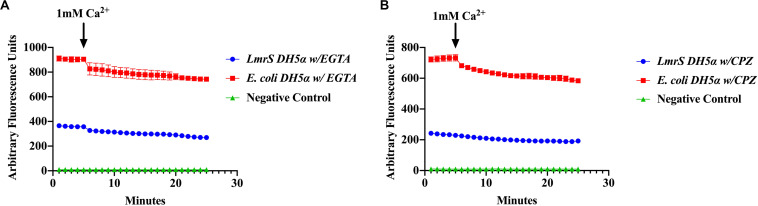
The calcium chelator EGTA **(A)** and the phenothiazine chlorpromazine (CPZ) **(B)** inhibit efflux of EtBr by *LmrS.* Recombinant *E. coli* cells and *E. coli* cells without the *LmrS* gene (used as control) were assayed by direct EtBr assay as described in the “Materials and Methods” section. The results represent the mean of at least three biological replicates. Each time point represents the average mean ± SD. A Kruskal–Wallis test was utilized to detect significant differences between the Ca^2+^ inhibitor treatments and without inhibitors. Significant differences were observed in both the *E. coli* DH5α and the recombinant *E. coli* strain as well as significant differences in efflux between the strains (*p* < 0.0001).

The effect of the Ca^2+^ chelator EGTA and the phenothiazine chlorpromazine (CPZ) were also evaluated as described in “Materials and Methods.” The cells were pre-incubated in the Ca^2+^ chelator, EGTA (Figure 7A) and the inhibitor, CPZ (Figure 7B) which resulted in an increased accumulation and higher fluorescence in both the wild-type and recombinant *E. coli* cells when compared to Figure 6 where no Ca^2+^ inhibitors or chelators were added. A Kruskal–Wallis analysis indicated significant efflux differences between the recombinant and wild-type *E. coli* cells (Figure 7). The ability of the recombinant *E. coli* cells to efflux EtBr more efficiently was indicated by the significant decrease in fluorescence as compared to the wild-type *E. coli.* After 5 min, 1 mM of Ca^2+^ was injected into the media and a decrease in fluorescence was observed in both the recombinant and wild-type *E. coli* cells indicating an enhancing effect on efflux.

## Discussion

The results presented in this study demonstrate that *S. aureus* cells maintain a tight control of cytosolic [Ca^2+^]i, which is a pre-requisite for its cell signaling. We also present evidence that various antibiotics induce Ca^2+^ mediated responses exhibiting unique and diverse spatial and temporal patterns. Moreover, here we demonstrate for the first time, that extrusion of EtBr by the multidrug transporter LmrS is enhanced by Ca^2+^.

Measurements of intracellular Ca^2+^ have been documented in several genera of bacteria but here we report for the first time, cytosolic Ca^2+^ measurements in an important pathogen, *S. aureus* (Domínguez, 2004; Domínguez et al., 2011). In agreement with previous studies, our results show that *S. aureus* maintains [Ca^2+^]i in the micromolar range in the presence of 0.5–5 mM external CaCl_2_. *S. aureus* cells responded rapidly to increases in the external Ca^2+^ concentration, while addition of the Ca^2+^ ion chelator EGTA produced a sharp decline. The maintenance of the low cytosolic Ca^2+^ is not only required by all cell types to protect them from the toxic effects of high cytosolic Ca^2+^ but also, to be able to use Ca^2+^ as a cellular signal. Any increase in cytosolic Ca^2+^ due to the transmission of the signal, must disappear quickly in order for the next signal to occur (Carafoli, 1987; Torrecilla et al., 2000).

The observation that Ca^2+^ transients increased significantly as pH values increased (from 5, to 7 to 9) suggests that *S. aureus* cells sense pH environmental conditions through changes in cytosolic Ca^2+^. These signals are essential for the organism to adapt and survive under wide variety of conditions, in the human host, during infection and colonization and in different environments. pH plays an important role in skin colonization, wound healing and immune cell chemotaxis. Moreover, the phagosome-lysosome environment is highly acidic. *S. aureus* must have sensor systems in response to the acidic conditions to adjust gene expression for survival (Venditri et al., 2003; Martins et al., 2008). Most Ca^2+^ exchangers in prokaryotes utilize Ca^2+^/H^+^ and Ca/Na^+^ gradients to transport Ca^2+^ into the cell via an electrogenic mechanism (Domínguez et al., 2015). The short Ca^2+^ transient (influx) developed at pH 5 (9.5 μM Ca^2+^) suggests proton competition for translocation of Ca^2+^ into the cell compared to pH 9 (23 μM Ca^2+^). These findings are in agreement with those presented by Naseem et al. (2008) in *E. coli* where a pH of 5 resulted in lowest luminescence. Our Ca^2+^ efflux results, however, differ slightly to those reported by Naseem et al. (2008). While Ca^2+^ decline was fast for the three pHs, at pH 9, Ca^2+^ levels did not reached basal levels compared to pH 5 and 7. It is interesting to note that pH triggers gene expression in various bacteria (Weinrick et al., 2004; Perez and Groisman, 2007; Martins et al., 2009; Serra-Cardona et al., 2015) including genes involved in cell envelope structure, ion transporters and multidrug transporters (Tucker et al., 2002; Weinrick et al., 2004; Hayes et al., 2006; Truong-Bolduc et al., 2011). However, the regulatory pathways by which bacteria sense and respond to pH stimuli have not been elucidated. The work presented here is of significance as it strongly suggests a role of Ca^2+^ in the regulation of genes encoding bacterial transporters or other proteins affecting efflux pumps in the response to antibiotics.

Exposure of different types of antibiotics to *S. aureus*-aequorin cells elicited immediate [Ca^2+^]i transients with unique properties for each antibiotic suggesting that *S. aureus* cells have the ability to sense and distinguish different classes of antibiotics. Interestingly, antibiotics within the same group such as gentamicin and kanamycin (aminoglycosides) showed different transients. Rapid increases in cytosolic Ca^2+^ in response to environmental pollutants and antibiotics have also been reported in *Saccharomyces cerevisiae* (Ruta et al., 2014; Farcasanu et al., 2018) and in the Cyanobacteria *Anabaena*, which also triggered specific Ca^2+^ signatures (Barrán-Berdón et al., 2011; González-Pleiter et al., 2017).

As previously stated, Ca^2+^ plays a pivotal role in many metabolic pathways including transport systems and are essential for signaling. The antimicrobial properties of the phenothiazines have been described since 1930 and 1940 (Grimsey and Piddock, 2019). The ways by which phenothiazines exert their antimicrobial effect include, damaging cell membranes, inhibiting Ca^2+^-dependent processes, disrupting cation-dependent transporters, acting as antagonist of Ca^2+^-binding proteins and in other ways (Grimsey and Piddock, 2019). Therefore, we investigated the effect of Ca^2+^ on the efflux of EtBr first by analyzing the effect of various divalent metals (Supplementary Material). CaCl_2_ showed a significantly greater effect on EtBr efflux compared to KCl_2_, MgCl_2_, and ZnCl_2_. Further evidence of the effect of Ca^2+^ on EtBr efflux was investigated by the addition of Ca^2+^ chelators, EGTA (higher specificity for Ca^2+^ binding) and EDTA, various efflux pump and Ca^2+^ binding proteins inhibitors such as the phenothiazines CPZ and TFP, which all had a strong inhibitory effect on efflux and accumulation of EtBr. The reversal of efflux inhibition by addition of Ca^2+^, indicated by the levels of fluorescence, showed the significant role that Ca^2+^ play in *S. aureus* efflux systems. These results are consistent with previous studies (Kaatz et al., 2003; Martins et al., 2009, 2011) and may have an impact in the development of novel inhibitors to combat antibiotic resistance.

Efflux transport systems are required for *S. aureus* survival in a wide range of environmental conditions, including high and low pH systems (Truong-Bolduc et al., 2011; Dastgheyb et al., 2015; Rippke et al., 2018). The efflux of EtBr was measured at different pHs (5, 7, and 9). The efflux of EtBr showed significant differences between the pHs and when glucose was added. In general, protons contributed to the ability of *S. aureus* to efflux EtBr. These results agree with those reported by Couto et al. (2008), Martins et al. (2011), which demonstrated differences in efflux due to pH and showed that proton-motive force is a significant contributor to efflux in *E. coli*. This was also observed in the present study as significant accumulation of EtBr was observed when CCCP, a proton un-coupler, was added at pH 5.

Cloning of the *lmrS* gene into *E. coli* conferred resistance to all antibiotics tested as compared to *E. coli* cells without the gene and efflux of EtBr was higher in *E. coli* cells carrying the *lmrS* gene than control cells. The effects of Ca^2+^ enhancing efflux of EtBr were corroborated in *E. coli-lmrS* by addition of Ca^2+^ and Ca^2+^ inhibitors consistent with our findings in *S. aureus* cells.

In conclusion these findings show that Ca^2+^homeostasis is maintained in *S. aureus* cells, that antibiotics induce a unique Ca^2+^ mediated response through transients in cytosolic Ca^2+^ and that the LmrS efflux pump is enhanced by Ca^2+^. Certainly, new therapeutic approaches are urgently needed to reduce the demand for antibiotics and to combat antimicrobial resistance. These studies suggest that further analysis of the effect of Ca^2+^ on efflux pump regulators, elucidation of their mechanism of action and control of their gene expression might lead to the development of novel efflux pump inhibitors, which could mitigate the antimicrobial resistance crisis.

## Data Availability Statement

The raw data supporting the conclusions of this article will be made available by the authors, without undue reservation.

## Ethics Statement

This study was approved by The University of Texas at El Paso (UTEP) Institutional Biosafety Committee and all protocols were done according to the rules and regulations of the Environmental and Safety Office at UTEP. Project # 1104408-4.

## Author Contributions

DD and AN: conceptualization and design. DD: administration and coordination. AN, NM, and AS: experimental work. AN and DD: data analysis and interpretation, writing, and editing. All authors contributed to the article and approved the submitted version.

## Conflict of Interest

The authors declare that the research was conducted in the absence of any commercial or financial relationships that could be construed as a potential conflict of interest.

## References

[B1] Alcalde-Rico M. Hernando-Amado S. Blanco P. Martínez J. L. (2016). Multidrug efflux pumps at the crossroad between antibiotic resistance and bacterial virulence. *Front. Microbiol.* 7:1483. 10.3389/fmicb.2016.01483 27708632 PMC5030252

[B2] Amaral L. Martins A. Molnar J. Kristiansen J. E. Martins M. Viveiros M. (2010). Phenothiazines, bacterial efflux pumps and targeting the macrophage for enhanced killing of intracellular XDRTB. *In Vivo* 24 409–424. 20668307

[B3] Arrizubieta M. J. Toledo-Arana A. Amorena B. Penadés J. R. Lasa I. (2004). Calcium inhibits bap-dependent multicellular behavior in *Staphylococcus aureus*. *J. Bacteriol.* 186 7490–7498. 10.1128/JB.186.22.7490-7498.2004 15516560 PMC524893

[B4] Asmat T. M. Tenenbaum T. Jonsson A. B. Schwerk C. Schroten H. (2014). Impact of calcium signaling during infection of *Neisseria meningitidis* to human brain microvascular endothelial cells. *PLoS One* 9:e114474. 10.1371/journal.pone.0114474 25464500 PMC4252121

[B5] Barrán-Berdón A. L. Rodea-Palomares I. Leganés F. Fernández-Piñas F. (2011). Free Ca^2 +^ as an early intracellular biomarker of exposure of cyanobacteria to environmental pollution. *Anal. Bioanal. Chem.* 400, 1015–1029. 10.1007/s00216-010-4209-3 20886207

[B6] Berridge M. J. Bootman M. D. Roderick H. L. (2003). Calcium signalling: dynamics, homeostasis and remodelling. *Nat. Rev. Mol. Cell Biol.* 4 517–529. 10.1038/nrm1155 12838335

[B7] Bishai W. R. Diarra B. Maiga M. Cohen K. A. Winglee K. Gupta S. (2013). Efflux inhibition with verapamil potentiates bedaquiline in *Mycobacterium tuberculosis*. *Antimicrob. Agents Chemother.* 58 574–576. 10.1128/aac.01462-13 24126586 PMC3910722

[B8] Broder U. N. Jaeger T. Jenal U. (2016). LadS is a calcium-responsive kinase that induces acute-to-chronic virulence switch in *Pseudomonas aeruginosa*. *Nat. Microbiol.* 2:16184. 10.1038/nmicrobiol.2016.184 27775685

[B9] Bruni G. N. Weekley R. A. Dodd B. J. T. Kralj J. M. (2017). Voltage-gated calcium flux mediates *Escherichia coli* mechanosensation. *Proc. Natl. Acad. Sci. U.S.A.* 114 9445–9450. 10.1073/pnas.1703084114 28808010 PMC5584419

[B10] Campbell A. K. (2015). *Intracellular Calcium.* Chichester: Wiley.

[B11] Campbell A. K. (2018). *The Ca^2 +^ Pump of Plasma Membranes.* Boca Raton, FL: CRC Revivals.

[B12] Carafoli E. (1987). Intracellular calcium homeostasis. *Annu. Rev. Biochem.* 56 395–433. 10.1146/annurev.bi.56.070187.002143 3304139

[B13] Carafoli E. Krebs J. (2016). Why calcium? How calcium became the best communicator. *J. Biol. Chem.* 291 20849–20857. 10.1074/jbc.R116.735894 27462077 PMC5076498

[B14] Chitsaz M. Brown M. H. (2017). The role played by drug efflux pumps in bacterial multidrug resistance. *Essays Biochem.* 61 127–139. 10.1042/EBC20160064 28258236

[B15] Clapham D. (1995). Calcium signaling. *Cell.* 80 259–268. 10.1016/0092-8674(95)90408-5 7834745

[B16] Conceição T. Coelho C. De Lencastre H. Aires-De-Sousa M. (2016). High prevalence of biocide resistance determinants in *Staphylococcus aureus* isolates from three African countries. *Antimicrob. Agents Chemother.* 60 678–681. 10.1128/AAC.02140-15 26552979 PMC4704190

[B17] Corrigan R. M. Foster T. J. (2009). An improved tetracycline-inducible expression vector for *Staphylococcus aureus*. *Plasmid* 61 126–129. 10.1016/j.plasmid.2008.10.001 18996145

[B18] Couto I. Costa S. S. Viveiros M. Martins M. Amaral L. (2008). Efflux-mediated response of *Staphylococcus aureus* exposed to ethidium bromide. *J. Antimicrob. Chemother.* 62, 504–513. 10.1093/jac/dkn217 18511413

[B19] Dastgheyb S. S. Villaruz A. E. Le K. Y. Tan V. Y. Duong A. C. Chatterjee S. S. (2015). Role of phenol-soluble modulins in formation of *Staphylococcus aureus* biofilms in synovial fluid. *Infect. Immun.* 83 2966–2975. 10.1128/IAI.00394-15 25964472 PMC4468530

[B20] Domínguez D. C. (2004). Calcium signalling in bacteria. *Mol. Microbiol.* 54 291–297. 10.1111/j.1365-2958.2004.04276.x 15469503

[B21] Domínguez D. C. Lopes R. Holland I. B. Campbell A. K. (2011). Proteome analysis of *B. subtilis* in response to calcium. *J. Analyt. Bioanalyt. Tech.* 6 1–9. 10.4172/2155-9872.s6-001

[B22] Domínguez D. C. Guragain M. Patrauchan M. (2015). Calcium binding proteins and calcium signaling in prokaryotes. *Cell Calcium* 57 151–165. 10.1016/j.ceca.2014.12.006 25555683

[B23] Du D. Wang-Kan X. Neuberger A. van Veen H. W. Pos K. M. Piddock L. J. V. (2018). Multidrug efflux pumps: structure, function and regulation. *Nat. Rev. Microbiol.* 16 523–539. 10.1038/s41579-018-0048-6 30002505

[B24] Eichstaedt S. Gäbler K. Below S. Müller C. Kohler C. Engelmann S. (2009). Effects of *Staphylococcus aureus*-hemolysin A on calcium signalling in immortalized human airway epithelial cells. *Cell Calcium* 45 165–176. 10.1016/j.ceca.2008.09.001 18922576

[B25] Esposito S. Garau J. David M. Z. Peters G. Lina G. Gould I. M. (2011). New insights into meticillin-resistant *Staphylococcus aureus* (MRSA) pathogenesis, treatment and resistance. *Int. J. Antimicrob. Agents* 39 96–104. 10.1016/j.ijantimicag.2011.09.028 22196394

[B26] Farcasanu I. C. Popa C.-V. Ruta L. L. (2018). “Calcium and cell response to heavy metals: can yeast provide an answer?,” in *Calcium and Signal Transduction*, eds Buchholz J. N. Behringer E. J. (Rijeka: IntechOpen). 10.5772/intechopen.78941

[B27] Fishman M. R. Giglio K. Fay D. Filiatrault M. J. (2018). Physiological and genetic characterization of calcium phosphate precipitation by *Pseudomonas* species. *Sci. Rep.* 8:10156. 10.1038/s41598-018-28525-4 29976945 PMC6033914

[B28] Floyd J. L. Smith K. P. Kumar S. H. Floyd J. T. Varela M. F. (2010). LmrS is a multidrug efflux pump of the major facilitator superfamily from *Staphylococcus aureus*. *Antimicrob. Agents Chemother.* 54 5406–5412. 10.1128/AAC.00580-10 20855745 PMC2981259

[B29] Foster T. J. (2017). Antibiotic resistance in *Staphylococcus aureus*. Current status and future prospects. *FEMS Microbiol. Rev.* 41 430–449. 10.1093/femsre/fux007 28419231

[B30] Fraunholz M. Sinha B. (2012). Intracellular *Staphylococcus aureus*: live-in and let die. *Front. Cell. Infect. Microbiol.* 2:43. 10.3389/fcimb.2012.00043 22919634 PMC3417557

[B31] González-Pleiter M. Leganés F. Fernández-Piñas F. (2017). Intracellular free Ca^2 +^ signals antibiotic exposure in cyanobacteria. *RSC Adv.* 7 35385–35393. 10.1039/c7ra03001k

[B32] Grimsey E. M. Piddock L. J. V. (2019). Do phenothiazines possess antimicrobial and efflux inhibitory properties? *FEMS Microbiol. Rev.* 43, 577–590. 10.1093/femsre/fuz017 31216574

[B33] Guragain M. Campbell A. Patrauchan M. (2016). Measurement of intracellular calcium concentration in *Pseudomonas aeruginosa*. *Bio-protocol* 6:e2041 10.21769/bioprotoc.2041

[B34] Guragain M. Lenaburg D. L. Moore F. S. Reutlinger I. Patrauchan M. A. (2013). Calcium homeostasis in *Pseudomonas aeruginosa* requires multiple transporters and modulates swarming motility. *Cell Calcium* 54 350–361. 10.1016/j.ceca.2013.08.004 24074964 PMC4410972

[B35] Hassan K. Elbourne L. Li L. Hewawasam Gamage H. Liu Q. Jackson S. (2015). An ace up their sleeve: a transcriptomic approach exposes the AceI efflux protein of *Acinetobacter baumannii* and reveals the drug efflux potential hidden in many microbial pathogens. *Front. Microbiol.* 6:333. 10.3389/fmicb.2015.00333 25954261 PMC4406071

[B36] Hassan K. A. Jackson S. M. Penesyan A. Patching S. G. Tetu S. G. Eijkelkamp B. A. (2013). Transcriptomic and biochemical analyses identify a family of chlorhexidine efflux proteins. *Proc. Natl. Acad. Sci. U.S.A.* 110 20254–20259. 10.1073/pnas.1317052110 24277845 PMC3864336

[B37] Hayes E. T. Wilks J. C. Sanfilippo P. Yohannes E. Tate D. P. Jones B. D. (2006). Oxygen limitation modulates pH regulation of catabolism and hydrogenases, multidrug transporters, and envelope composition in *Escherichia coli* K-12. *BMC Microbiol.* 6:89. 10.1186/1471-2180-6-89 17026754 PMC1626474

[B38] Herbaud M. L. Guiseppi A. Denizot F. Haiech J. Kilhoffer M. C. (1998). Calcium signalling in *Bacillus subtilis*. *Biochim. Biophys. Acta* 1448 212–226. 10.1016/S0167-4889(98)00145-1 9920412

[B39] Jang S. (2016). Multidrug efflux pumps in *Staphylococcus aureus* and their clinical. *J. Microbiol.* 54 1–8. 10.1007/s12275-016-5159-z 26727895

[B40] Jones H. E. Holland I. B. Baker H. L. Campbell A. K. (1999). Slow changes in cytosolic free Ca^2 +^ in *Escherichia Coli* highlight two putative influx mechanisms in response to changes in extracellular calcium. *Cell Calcium* 25 265–274. 10.1054/ceca.1999.0028 10378087

[B41] Kaatz G. W. Moudgal V. V. Seo S. M. Kristiansen E. Kristiansen J. E. (2003). Phenothiazines and thioxanthenes inhibit multidrug efflux pump activity in *Staphylococcus aureus* phenothiazines and thioxanthenes inhibit multidrug efflux pump activity in *Staphylococcus aureus*. *Antimicrob. Agents Chemother.* 47 719–726. 10.1128/AAC.47.2.719 12543683 PMC151737

[B42] King M. M. Kayastha B. B. Franklin M. J. Patrauchan M. A. (2020). Calcium regulation of bacterial virulence. *Adv. Exp. Med. Biol.* 1131, 827–855. 10.1007/978-3-030-12457-1_33 31646536 PMC7473484

[B43] Knight M. R. Campbell A. K. Smith S. M. Trewavas A. J. (1991). Transgenic plant aequorin reports the effects of touch and cold-shock and elicitors on cytoplasmic calcium. *Nature* 352 524–526. 10.1038/352524a0 1865907

[B44] Kobayashi S. D. Malachowa N. Deleo F. R. (2015). Pathogenesis of *Staphylococcus aureus* abscesses. *Am. J. Pathol.* 185 1518–1527. 10.1016/j.ajpath.2014.11.030 25749135 PMC4450319

[B45] Koul S. Somayajulu A. Advani M. J. Reddy H. (2009). A novel calcium binding protein in mycobacterium tuberculosis – potential target for trifluoperazine. *Indian J. Exp. Biol.* 47 480–488. 19639701

[B46] Li J. Zhao X. Tian X. Li J. Sjollema J. Wang A. (2015). Retention in treated wastewater affects survival and deposition of *Staphylococcus aureus* and *Escherichia coli* in sand columns. *Appl. Environ. Microbiol.* 81 2199–2205. 10.1128/AEM.03740-14 25595758 PMC4345387

[B47] Martins A. MacHado L. Costa S. Cerca P. Spengler G. Viveiros M. (2011). Role of calcium in the efflux system of *Escherichia coli*. *Int. J. Antimicrob. Agents* 37 410–414. 10.1016/j.ijantimicag.2011.01.010 21419607

[B48] Martins A. Spengler G. Rodrigues L. Viveiros M. Ramos J. Martins M. (2009). pH modulation of efflux pump activity of multi-drug resistant *Escherichia coli*: protection during its passage and eventual colonization of the colon. *PLoS One* 4:e6656. 10.1371/journal.pone.0006656 19684858 PMC2722724

[B49] Martins M. Viveiros M. Amaral L. Costa S. Couto I. (2008). Efflux-mediated response of *Staphylococcus aureus* exposed to ethidium bromide. *J. Antimicrob. Chemother.* 62 504–513. 10.1093/jac/dkn217 18511413

[B50] Moretti C. Trabalza S. Granieri L. Caballo-Ponce E. Devescovi G. Del Pino A. M. (2019). A Na^+^/Ca^2 +^ exchanger of the olive pathogen *Pseudomonas savastanoi* pv. *savastanoi* is critical for its virulence. *Mol. Plant Pathol.* 20 716–730. 10.1111/mpp.12787 30912619 PMC6637891

[B51] Naseem R. Holland I. B. Jacq A. Wann K. T. Campbell A. K. (2008). pH and monovalent cations regulate cytosolic free Ca^2 +^ in *E. coli*. *Biochim. Biophys. Acta* 1778 1415–1422. 10.1016/j.bbamem.2008.02.006 18342619

[B52] Naseem R. Wann K. T. Holland I. B. Campbell A. K. (2009). ATP regulates calcium efflux and growth in *E. coli*. *J. Mol. Biol.* 391 42–56. 10.1016/j.jmb.2009.05.064 19481094

[B53] Nichols R. J. Sen S. Choo Y. J. Beltrao P. Zietek M. Chaba R. (2011). Phenotypic landscape of a bacterial cell. *Cell* 144 143–156. 10.1016/j.cell.2010.11.052 21185072 PMC3060659

[B54] Onyango L. A. Alreshidi M. M. (2018). Adaptive metabolism in staphylococci: survival and persistence in environmental and clinical settings. *J. Pathog.* 2018:1092632. 10.1155/2018/1092632 30327733 PMC6171259

[B55] Perez J. C. Groisman E. A. (2007). Acid pH activation of the PmrA/PmrB two-component regulatory system of *Salmonella enterica*. *Mol. Microbiol.* 63 283–293. 10.1111/j.1365-2958.2006.05512.x 17229213 PMC1804205

[B56] Piddock L. J. V. (2006). Multidrug-resistance efflux pumps? not just for resistance. *Nat. Rev. Microbiol.* 4 629–636. 10.1038/nrmicro1464 16845433

[B57] Pule C. M. Sampson S. L. Warren R. M. Black P. A. van Helden P. D. Victor T. C. (2016). Efflux pump inhibitors: targeting mycobacterial efflux systems to enhance TB therapy. *J. Antimicrob. Chemother.* 71 17–26. 10.1093/jac/dkv316 26472768

[B58] Rajagopal S. Murugavel P. (2017). *Calcium Signaling: From Physiology to Diseases.* Singapore: Springer 10.1007/978-981-10-5160-9

[B59] Rippke F. Berardesca E. Weber T. M. (2018). PH and microbial infections. *Curr. Prob. Dermatol.* 54 87–94. 10.1159/000489522 30130777

[B60] Romero D. Traxler M. F. López D. Kolter R. (2011). Antibiotics as signal molecules. *Chem. Rev.* 111 5492–5505. 10.1021/cr2000509 21786783 PMC3173521

[B61] Ruta L. L. Popa V. C. Nicolau I. Danet A. F. Iordache V. Neagoe A. D. (2014). Calcium signaling mediates the response to cadmium toxicity in Saccharomyces cerevisiae cells. *FEBS Lett.* 588 3202–3212. 10.1016/j.febslet.2014.07.001 25017440

[B62] Santos Costa S. Viveiros M. Amaral L. Couto I. (2013). Multidrug efflux pumps in *Staphylococcus aureus*: an update. *Open Microbiol J.* 7 59–71. 10.1111/avsc.12262 23569469 PMC3617543

[B63] Sapula S. A. Brown M. H. (2016). “Antimicrobial drug efflux pumps in *Staphylococcus aureus*,” in *Efflux-Mediated Antimicrobial Resistance in Bacteria: Mechanisms, Regulation and Clinical Implications*, eds Li X.-Z. Elkins C. A. Zgurskaya H. I. (Cham: Springer International Publishing), 165–195. 10.1007/978-3-319-39658-3_7

[B64] Sarkisova S. Patrauchan M. A. Berglund D. Nivens D. E. Franklinm M. J. (2005). Calcium-induced virulence factors associated with the extracellular matrix of mucoid *Pseudomonas aeruginosa* biofilms. *J. Bacteriol.* 187 4327–4337. 10.1128/JB.187.13.4327-4337.2005 15968041 PMC1151780

[B65] Sarkisova S. A. Lotlikar S. R. Guragain M. Kubat R. Cloud J. Franklin M. J. (2014). A *Pseudomonas aeruginosa* EF-hand protein, EfhP (PA4107), modulates stress responses and virulence at high calcium concentration. *PLoS One* 9:e98985. 10.1371/journal.pone.0098985 24918783 PMC4053335

[B66] Schindler B. D. Jacinto P. L. Buensalido J. A. L. Seo S. M. Kaatz G. W. (2015). Clonal relatedness is a predictor of spontaneous multidrug efflux pump gene overexpression in *Staphylococcus aureus*. *Int. J. Antimicrob. Agents* 45 464–470. 10.1016/j.ijantimicag.2014.11.007 25548027

[B67] Schindler B. D. Kaatz G. W. (2016). Multidrug efflux pumps of Gram-positive bacteria. *Drug Resist. Updat.* 27 1–13. 10.1016/j.drup.2016.04.003 27449594

[B68] Serra-Cardona A. Canadell D. Ariño J. (2015). Coordinate responses to alkaline pH stress in budding yeast. *Microb. Cell* 2 182–196. 10.15698/mic2015.06.205 28357292 PMC5349140

[B69] Takahashi H. Kopriva S. (2019). Sulfate transport systems in plants: Functional diversity and molecular mechanisms underlying regulatory coordination. *J. Exp. Bot.* 70 4075–4087. 10.1093/jxb/erz132 30907420

[B70] Thomas V. L. Sanford B. A. Ramsay M. A. (1993). Calcium- and mucin-binding proteins of staphylococci. *J. Gen. Microbiol.* 139 623–629. 10.1099/00221287-139-3-623 8473868

[B71] Torrecilla I. Leganés F. Bonilla I. Fernández-Piñas F. (2000). Use of recombinant aequorin to study calcium homeostasis and monitor calcium transients in response to heat and cold shock in cyanobacteria. *Plant Physiol.* 123 161–176. 10.1104/pp.123.1.161 10806234 PMC58991

[B72] Torrecilla I. Leganés F. Bonilla I. Fernández-Piñas F. (2001). Calcium transients in response to salinity and osmotic stress in the nitrogen-fixing cyanobacterium *Anabaena* sp. PCC7120, expressing cytosolic apoaequorin. *Plant Cell Environ.* 24 641–648. 10.1046/j.0016-8025.2001.00708.x

[B73] Truong-Bolduc Q. C. Bolduc G. R. Okumura R. Celino B. Bevis J. Liao C. H. (2011). Implication of the NorB efflux pump in the adaptation of *Staphylococcus aureus* to growth at acid ph and in resistance to moxifloxacin. *Antimicrob. Agents Chemother.* 55 3214–3219. 10.1128/AAC.00289-11 21555767 PMC3122426

[B74] Tucker D. L. Tucker D. L. Tucker N. Tucker N. Conway T. Conway T. (2002). Gene expression profling of the pH response in *Escherichia coli*. *J. Bacteriol.* 184 6551–6558. 10.1128/JB.184.23.6551 12426343 PMC135413

[B75] Van Nhieu G. T. Clair C. Bruzzone R. Mesnil M. Sansonetti P. Combettes L. (2003). Connexin-dependent inter-cellular communication increases invasion and dissemination of *Shigella* in epithelial cells. *Nat. Cell Biol.* 5 720–726. 10.1038/ncb1021 12844145

[B76] Venditri M. Falcone M. Micozzi A. Carfagna P. Taglietti F. Serra P. F. (2003). *Staphylococcus aureus* bacteremia in patients with hematologic malignancies: a retrospective case-control study. *Haematologica* 88 923–930. 12935981

[B77] Webber M. A. Piddock L. J. V. (2003). The importance of efflux pumps in bacterial antibiotic resistance. *J. Antimicrob. Chemother.* 51 9–11. 10.1093/jac/dkg050 12493781

[B78] Weinrick B. Dunman P. M. McAleese F. Murphy E. Projan S. J. Fang Y. (2004). Effect of mild acid on gene expression in *Staphylococcus aureus*. *J. Bacteriol.* 186 8407–8423. 10.1128/JB.186.24.8407-8423.2004 15576791 PMC532443

[B79] Zampese E. Pizzo P. (2012). Intracellular organelles in the saga of Ca^2 +^ homeostasis: different molecules for different purposes? *Cell. Mol. Life Sci.* 69 1077–1104. 10.1007/s00018-011-0845-9 21968921 PMC11114864

[B80] Zhou Y. Xue S. Yang J. J. (2013). Calciomics: integrative studies of Ca^2 +^-binding proteins and their interactomes in biological systems. *Metallomics* 5 29–42. 10.1039/c2mt20009k 23235533 PMC3614492

