# Supplementary material for: Evidence of Calcium Signaling and Modulation of the LmrS Multidrug Resistant Efflux Pump Activity by Ca2 + Ions in S. aureus
Source: Front Microbiol. 2020 Oct 22;11:573388. doi: 10.3389/fmicb.2020.573388 (PMC7642317; doi:10.3389/fmicb.2020.573388)
Supplement: Supplementary file 1 [file Data_Sheet_1.pdf]

Original Data Set

Minutes	"*lmrS* Clone"	"*lmrS* Clone"	"*lmrS* Clone"	"*E. coli* control"	"*E. coli* control"	"E. coli control"
1	147	158.8712	161	246	245	249
2	149	159.1111	161	246	242	245
3	150	158.3146	159	245	242	244
4	148	157.0426	158	247	242	247
5	147	156.8048	157	248	245	247
6	148	157.4783	157	249	247	249
7	149	157.7013	158	248	244	245
8	149	158.7006	157	245	242	242
9	149	157.7192	158	245	241	242
10	150	157.4164	158	246	243	241
11	150	158.4405	158	246	244	242
12	149	157.6932	158	245	242	241
13	149	158.8472	157	246	244	241
14	151	158.7223	158	245	242	240
15	151	160.4564	159	244	240	241
16	150	160.9971	158	245	241	240
17	149	160.7805	159	246	242	239
18	150	159.8787	158	246	243	240
19	150	160.7781	159	244	242	239
20	151	161.1780	158	244	244	239

Normalized Data

Minutes	"*lmrS* Clone"	"*lmrS* Clone"	"*lmrS* Clone"	"*E. coli* control"	"*E. coli* control"	"E. coli control"
1.000	-2.052	1.638	2.300	0.650	0.500	1.100
2.000	-1.430	1.713	2.300	0.650	0.050	0.500
3.000	-1.119	1.465	1.678	0.500	0.050	0.350
4.000	-1.741	1.070	1.367	0.800	0.050	0.800
5.000	-2.052	0.996	1.056	0.950	0.500	0.800
6.000	-1.741	1.205	1.056	1.100	0.800	1.100
7.000	-1.430	1.274	1.367	0.950	0.350	0.500
8.000	-1.430	1.585	1.056	0.500	0.050	0.050
9.000	-1.430	1.280	1.367	0.500	-0.100	0.050
10.000	-1.119	1.186	1.367	0.650	0.200	-0.100
11.000	-1.119	1.504	1.367	0.650	0.350	0.050
12.000	-1.430	1.272	1.367	0.500	0.050	-0.100
13.000	-1.430	1.630	1.056	0.650	0.350	-0.100
14.000	-0.809	1.592	1.367	0.500	0.050	-0.250
15.000	-0.809	2.131	1.678	0.350	-0.250	-0.100
16.000	-1.119	2.299	1.367	0.500	-0.100	-0.250
17.000	-1.430	2.231	1.678	0.650	0.050	-0.400
18.000	-1.119	1.951	1.367	0.650	0.200	-0.250
19.000	-1.119	2.231	1.678	0.350	0.050	-0.400

20.000 -0.809 2.355 1.367 0.350 0.350 -0.400

Kolmogorov-Smirnov test

"Table Analyzed" "*E.coli* control vs *lmrS* clone Normalization Data"

"Column A" "*lmrS* Clone"
vs. vs.
"Column B" "*E. coli* control"

"Kolmogorov-Smirnov test"
" P value" 0.0015
" Exact or approximate p value?" Approximate
" P value summary" **
" Significantly different (P < 0.05)?" Yes
" Kolmogorov-Smirnov D" 0.6000

The original data set represents three biological replicates or sub-columns. Each strain was prepared and resuspended in PBS as described in the materials and methods. The experiments were conducted over a twenty minute timeframe. Each of the sub-columns were averaged and the means were normalized. 0% was defined as the lowest mean in each data set as well as y=0 (data not shown). 100% was defined as the largest mean in each data set or at a value above the fluorescent values of both strains. Both normalized data sets were analyzed using a Kolmogorov-Smirnov test. Significant differences were found between both data sets with an approximate P value of ($p<0.0015$).
