# Supplementary material for: Evidence of Calcium Signaling and Modulation of the LmrS Multidrug Resistant Efflux Pump Activity by Ca2 + Ions in S. aureus
Source: Front Microbiol. 2020 Oct 22;11:573388. doi: 10.3389/fmicb.2020.573388 (PMC7642317; doi:10.3389/fmicb.2020.573388)
Supplement: Supplementary file 2 [file Data_Sheet_2.pdf]

Supplemenatry data Metals Assay

We evaluated the effect of various divalent cations ($CaCl_2$, $ZnCl_2$, $MgCl_2$ and KCl at a concentration of 1 mM) on efflux of EtBr in *S. aureus* cells. Cells were pelleted and washed twice with PBS to remove excess BHI. They were then incubated for 30 minutes in EtBr and a divalent metal cation. Fluorescence was subsequently monitored for 20 min at 585 nm. The data indicated that $CaCl_2$ had a significantly greater (Kruskall-Wallis $p < 0.0001$) effect on the efflux of EtBr compared to the other metals and control (*S. aureus* cells and EtBr without metal)

Divalent cations assay- Supplementary Figure

S. aureus cells were incubated in 1mM of divalent metals, $MgCl_2$, $CaCl_2$, $ZnCl_2$, KCl and EtBr alone (control) for observation of efflux. Significant differences were observed between Ca^{2+} and other divalent metals as well as the control. Each time point represents the average mean +/- SD of at least three biological replicates and three independent experiments. A Kruskall-Wallis analysis was used to determine significant differences. ($p < 0.0001$).
