# Supplementary figures and images for: Evidence of Calcium Signaling and Modulation of the LmrS Multidrug Resistant Efflux Pump Activity by Ca2 + Ions in S. aureus
Source: Front Microbiol. 2020 Oct 22;11:573388. doi: 10.3389/fmicb.2020.573388 (PMC7642317; doi:10.3389/fmicb.2020.573388)

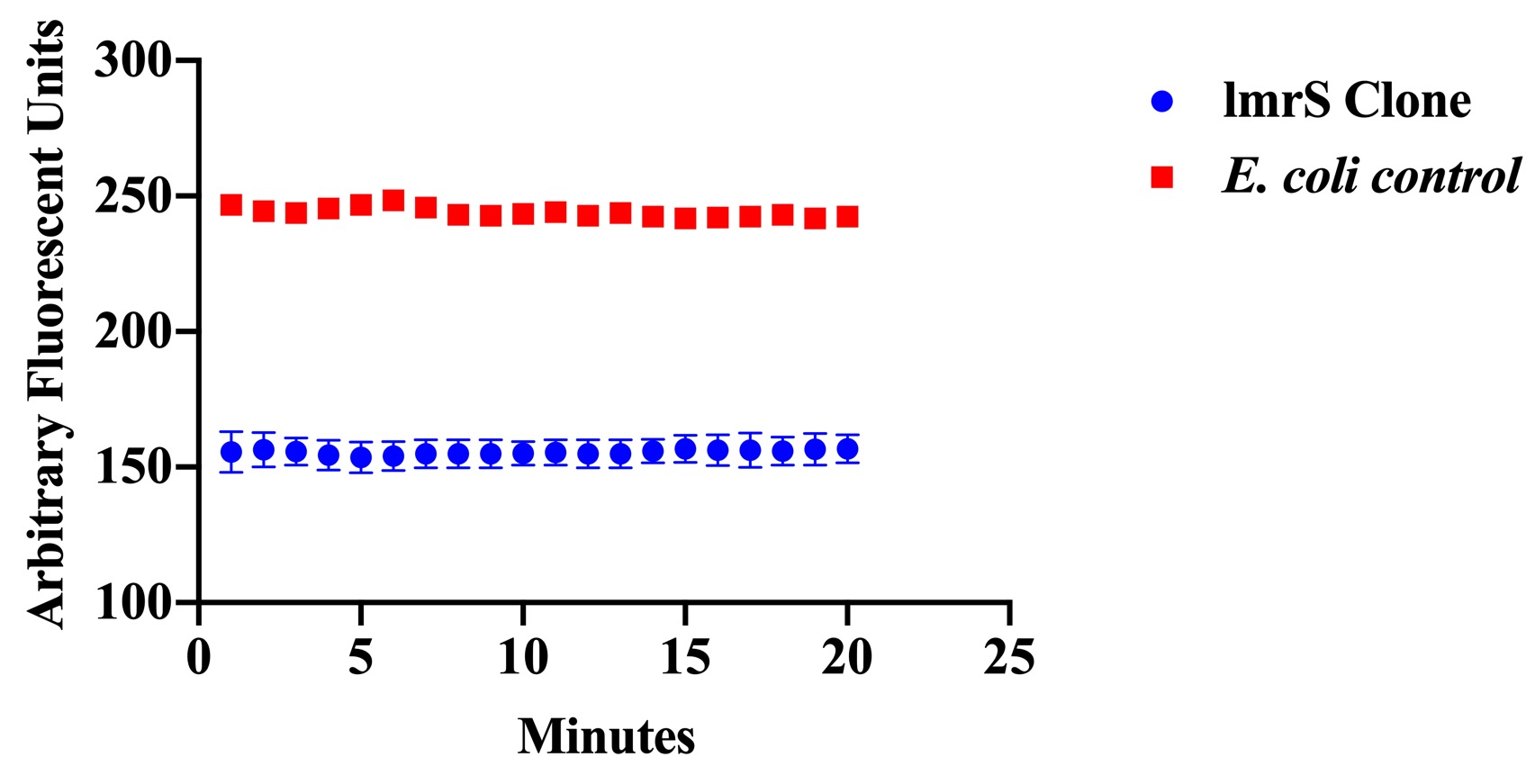

Supplement: Supplementary file 3 [file Image_1.jpg]

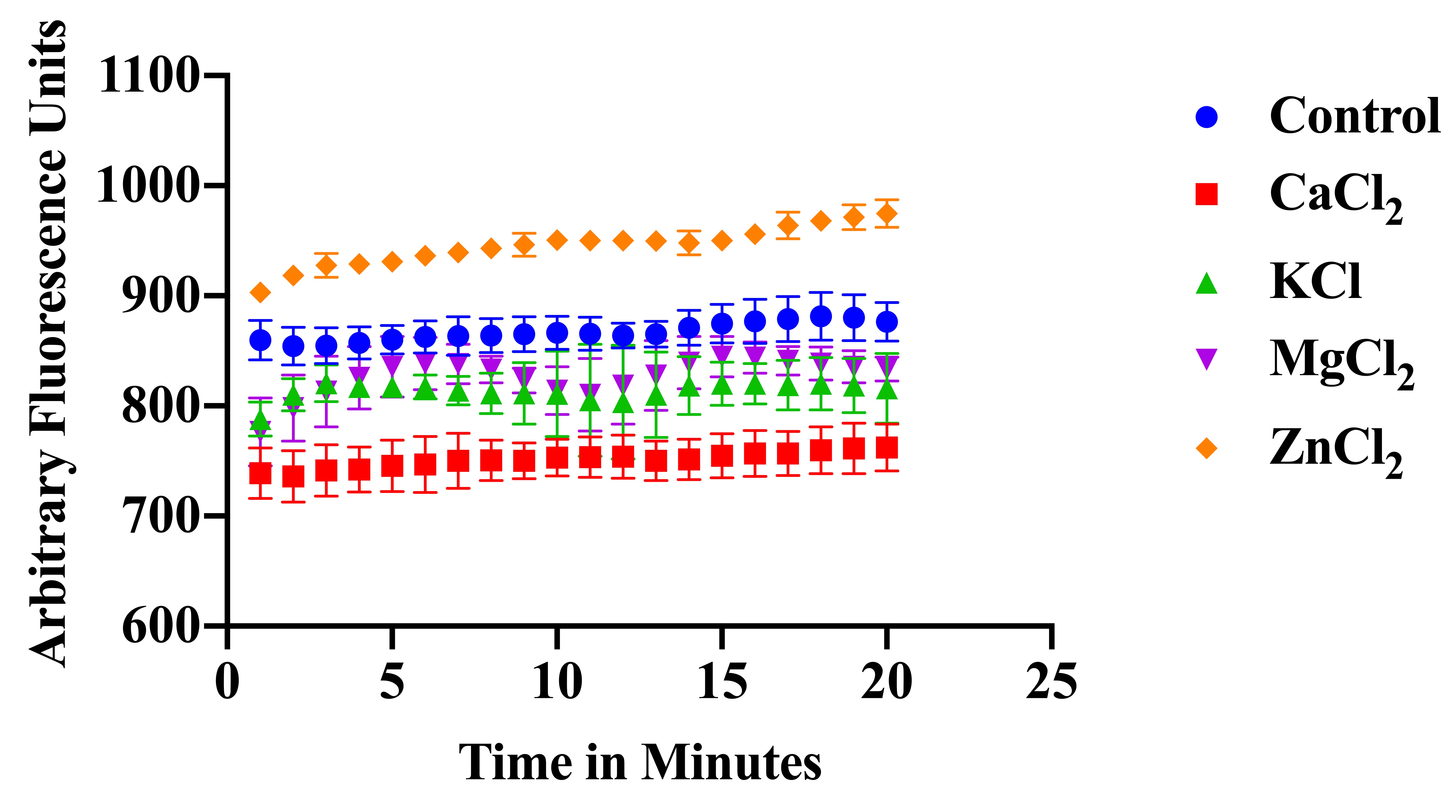

Supplement: Supplementary file 4 [file Image_2.jpg]
